# Peripheral blood lymphocytes subtypes as new predictors for neoadjuvant therapy efficacy in breast cancer

**DOI:** 10.1002/cam4.4666

**Published:** 2022-04-11

**Authors:** Jikun Feng, Jiarong Yi, Xiazi Zouxu, Jianxia Li, Zhenchong Xiong, Xinjian Huang, Wenjing Zhong, Weiling Huang, Feng Ye, Xi Wang

**Affiliations:** ^1^ Department of Breast Oncology, Sun Yat‐sen University Cancer Center, the State Key Laboratory of Oncology in South China Collaborative Innovation Center for Cancer Medicine Guangzhou Guangdong China; ^2^ Department of Medical Oncology, The Sixth Affiliated Hospital of Sun Yat‐sen University Guangdong Provincial Key Laboratory of Colorectal and Pelvic Floor Diseases Guangzhou China

**Keywords:** breast cancer, independent predictors, neoadjuvant therapy, PBLs subtypes

## Abstract

**Background:**

Host immunity plays an important role in tumor development and treatment. Tumor‐infiltrating lymphocytes (TILs) have been proven to predict the efficacy of neoadjuvant therapy (NAT) in breast cancer (BC) patients, but their application is limited due to various reasons. This study aims to explore the relationship between peripheral blood lymphocytes (PBLs) subsets distribution and the efficacy of NAT.

**Methods:**

Between December 2017 and March 2021, a total of 116 BC patients appropriate for NAT in Sun Yat‐Sen University cancer center were enrolled, pre‐NAC baseline blood samples were taken for further flow cytometry analysis to quantitatively evaluate the PBLs subsets distribution, and corresponding clinical information including pathological complete response (pCR) rate of NAT response were recorded.

**Results:**

Baseline CD3+ T cells(OR 1.11, 1.03–1.21, *p* = 0.011), CD8+ T cells (OR 1.09, 1.02–1.18, *p* = 0.015), and NK cells (OR 0.91, 0.83–0.98, *p* = 0.028) in PBLs subgroup distribution were independent predictors of pCR in BC patients receiving NAT, in which CD8+ T cells had the highest predictive ability (AUC = 0.76). Compared with some previous prediction indicators, its prediction ability has been improved to some extent.

**Conclusion:**

Peripheral baseline CD3+ T cells, CD8+ T cells, and NK cells were independent predictors of pCR in BC patients receiving NAT, in which CD8+ T cells had the highest predictive ability. Therefore, it can provide newly non‐invasive, relatively accurate and easily accessible predictors for corresponding patients, and help clinicians better understand tumor immunity.

## Graphical abstract

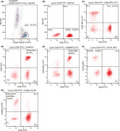

**Lay summary**

A retrospective study to find the correlation between PBLs subtypes and NAT efficacy, aiming to explore the independent predictors of pCR with great prediction ability.

## 1 INTRODUCTION

According to the latest cancer statistics, breast cancer (BC) has the annual incidence of 11.6%, which has been an important threat to women's health.^1^

Currently, neoadjuvant therapy (NAT) has become the standard treatment for patients with advanced intermediate BC, which can achieve the degradation of breast tumor and enable locally advanced patients to obtain the opportunity for mastectomy or breast conserving surgery.^2^ Pathological complete response (pCR) refers to the complete disappearance of the primary tumor after NAT(identified as Miller‐payne grade 5 together with negative lymphatic metastasis), which was an indicator of therapeutic efficacy.^2^ Furthermore, pCR is used as a prognostic factor for BC patients, for studies have shown that achievement of pCR after NAT was significantly correlated with longer disease‐free survival (DFS) and overall survival (OS).^3^, ^4^, ^5^

The relationship between host immune system and tumor is an hotspot in cancer research at present. Studies have shown that tumor infiltrated lymphocytes (TILs) is an independent predictor for prognosis and NAT efficacy in BC patients.^6^, ^7^, ^8^, ^9^, ^10^, ^11^ However, the clinical use of TILs has some limitations: First, there is spatial heterogeneity in the distribution of TILs, which could be affected by many factors, such as tumor cell growth patterns or histopathological types.^12^ For example, the TILs of tumors with solid growth pattern and dissociative growth pattern may be completely different.^13^ Therefore, for BC patients, there may be inevitable deviation in TILs evaluation of tissue specimens obtained by preoperative core needle aspiration to evaluate the efficacy of NAT.^14^ Second, about 16% of BC patients did not have TILs, for BC has been identified as “cold tumor”, which had less tumor immune infiltration than other tumors, such as colon cancer, lung cancer, and liver cancer.^15^ This makes it difficult to evaluate TILs, while PBLs can be easily detected in patients' peripheral blood anytime and anywhere. At present, some studies have indicated that PBLs subsets can predict the prognosis of BC patients,^16^, ^17^, ^18^ but there is no study on the prediction effect of PBLs subsets on NAT in BC patients.

Therefore, this study intends to explore the correlation between PBLs subgroups distribution and NAT efficacy in BC, and its ability to influence and predict pCR rates. Then explore and find new indicators to predict the efficacy of NAT in BC, and to make up for the current deficiency.

## 2 MATERIALS AND METHODS

### 2.1 Patients

From December 2017 to March 2021, a total of 116 patients' data were collected in Sun Yat‐Sen University Cancer Center. The diagnosis of invasive breast cancer was confirmed by preoperative core needle biopsy pathology. The main inclusion criteria were: 1. 18–80 years of age female patients; 2. Preoperative imaging diagnosed as the early and intermediate stage breast cancer, and eligibility for NAT after comprehensive evaluation according to the latest NCCN BC guideline. Exclusion criteria mainly were: 1. previous antitumor therapy, that is, surgery, chemotherapy, radiotherapy, targeted therapy, or immunotherapy, etc; 2. secondary tumors; 3. HIV infection or other immune system diseases; 4. past use of immune agents or drugs and health care products that may affect immune function; 5. state of inflammation and infection within nearly 1 week, including patients' blood white blood cells (WBC), neutrophils, and C‐reactive protein (CRP) that are out of the normal range. This study was approved by the Ethics Committee of Sun Yat‐Sen University Cancer Center.

### 2.2 General information and examination results

Patients' information was obtained from the breast cancer single disease research platform of Sun Yat‐Sen University Cancer Center. Inclusion and exclusion criteria were described above. General patients' information, histopathological findings, and specific blood test results were collected retrospectively. General patient information included age, gender, BMI. The histopathological data included baseline information before NAT: pathological type, tumor immunohistochemical staining, and postoperative paraffin pathological findings including pathological type and stage, Miller‐Payne stage score, metastatic status of axillary lymph nodes, immunohistochemical staining results. ER positivity was defined as ER ≥1%, PR positivity was defined as PR ≥1%, HER2 expression was confirmed by immunohistochemistry or fluorescence in situ hybridization.HR+HER2‐ BC was defined as ER/PR+ and HER2‐. HER2+ BC defined as HER2+,ER/PR+ or ‐ TNBC defied as ER, PR, and HER2‐ LMR defined as lymphocytes / monocytes ratio acquired with routine blood tests, NLR defined as neutrophils / lymphocytes ratio. pCR defined as pathological Miller‐payne 5 together with no lymph nodes metastasis after NAT.

### 2.3 Flow cytometry analysis of PBLs subtypes

One week within NAT treatment, 4~10 ml blood samples were collected from the above enrolled patients. Then within 30 min, 300 μl blood samples were coated with primary antibody CD3‐PC5/CD4‐FITC/CD8‐PE (IM1650), CD3‐FITC/(CD16þ/CD56)‐PE

(A07735), CD4‐FITC (A007750), CD8‐FITC (A07756), CD19‐PC5 (A07771), and CD25‐PE (A07774) at room temperature for 10 min, followed by centrifugation at 1300*g* at room temperature for 5 min. Remove the supernatant. Subsequently, 500 ul PBS was added into the sample, and flow‐cytometric analysis was performed on the machine Beckman‐Coulter FC500 (Beckman Coulter, Inc.). Analysis was performed using CXP analysis software (Beckman Coulter, Inc.). PBLs subgroups information was output in the percentage of total PBLs.

### 2.4 Statistical analysis

The chi‐squared test was used to assess the relationship between clinicopathological parameters and pCR. Two‐sided Wilcoxon test was used to detect difference of PBLs quantification between pCR and non‐PCR group. Logistic regression analysis was performed for univariate and multivariate analysis with an enter method. ROC curves were also plotted to verify the accuracy of peripheral blood immune markers (NLR, LMR, CD3, CD3CD4, CD3CD8, CD19, and CD16CD56) and clinicopathological markers (HER2, ER, PR, Ki67, T stage, and N stage) for pCR prediction. Statistical analysis was performed using R version 4.0.1 software and GraphPad Prism version 7.0 software. *p* < 0.05 was considered statistically significant.

## 3 RESULTS

### 3.1 General patient characteristics and histopathological findings

According to the above inclusion and exclusion criteria, a total of 116 patients were enrolled in this study. Corresponding analysis of the general characteristics of the patients showed that most of the patients were older than 40 years old (*n* = 92,79.3%), most of the patients had a BMI of less than or equal to 24 (*n* = 73,62.9%). According to preoperative clinical TNM evaluation stage, most patients presented with cT1‐2 (*n* = 85,73.3%), while *n*1 = 60 (51.7%) and *n*2 = 56 (48.3%) for patients with lymph node stage cN0‐1 and cN2‐3, respectively, patients were divided into HR+HER2‐ (*n* = 52,44.8%),HER2+ (*n* = 53,45.7%), and TNBC (*n* = 11,9.5%) by referring to the pathological immunohistochemical results of core needle biopsy before NAT. (Table1). After NAT, 19 (16.4%) patients achieved pCR.

**TABLE 1:** Clinicopathological parameters(*n* = 116)

Variables	Numbers	Percent(%)
Age (years)		
>40	92	79.3%
<=40	24	20.7%
BMI		
>24	43	37.1%
<=24	73	62.9%
cT stage		
T1‐2	85	73.3%
T3‐4	31	26.7%
cN stage		
N0‐1	60	51.7%
N2‐3	56	48.3%
Ki67		
>40	48	41.4%
<=40	68	58.6%
Molecular subtype		
HR+ HER2(−)	52	44.8%
HER2 (+)	53	45.7%
TNBC	11	9.5%

Abbreviation: BMI, body mass index; ER, estrogen receptor; PR, progesterone receptor; HER2, human epidermal growth factor receptor 2.HR+HER2‐, ER+ or PR+, HER2‐; HER2+, ER+/ PR+‐, HER2+; TNBC, ER‐, PR‐, HER2‐; cT stage, clinical tumor stage; cN stage, clinical lymph nodes stage.

We observed that pCR rate was significantly different in different molecular subgroups (HR+HER2‐, HER2+, and TNBC) (*p* = 0.02), and the pCR rate of HER2+ and TNBC BC was significantly higher than that of HR+HER2‐. However, other index such as age, BMI, tumor stage and Ki67, did not suggest a significant correlation with pCR in our cohort (Table2).

**TABLE 2:** Correlations between clinicopathologic factors and pCR rates

	pCR (*n* = 19)	Non‐pCR (*n* = 97)	*p*
>40	14	78	0.7246
<=40	5	19	
BMI > 24	7	36	1
BMI < =24	12	61	
T1‐2	16	69	0.3711
T3‐4	3	28	
N0‐1	10	50	1
N2‐3	9	47	
ER+	11	75	0.1384
ER‐	8	22	
PR+	9	64	0.2019
PR‐	10	33	
HER2+	13	40	0.0544
HER2‐	6	57	
Ki67 > 40	11	37	0.179
Ki67 < =40	8	60	
HR+HER2‐	3	49	0.020
HER2+	13	40	
TNBC	3	8	

Abbreviation: BMI, body mass index; ER, estrogen receptor; PR, progesterone receptor; HER2, human epidermal growth factor receptor 2.HR+HER2‐, ER+ or PR+, HER2‐; HER2+, ER+/ PR+‐, HER2+; TNBC, ER‐, PR‐, HER2‐;cT stage, clinical tumor stage; cN stage, clinical lymph nodes stage.

### 3.2 Relationship between PBLs subsets and NAT efficacy

**FIGURE 1:**
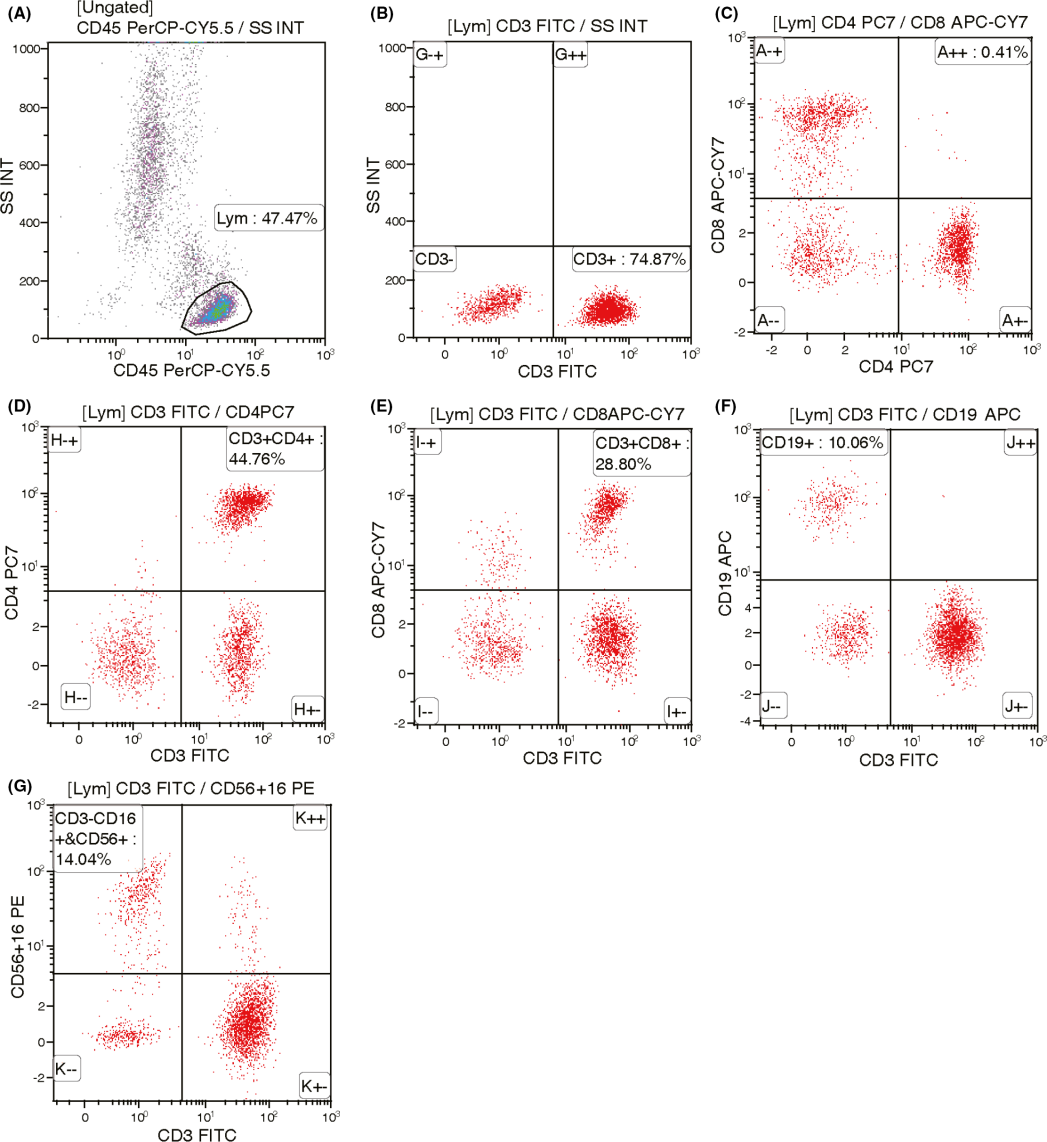
Flow cytometry of PBLs subtypes from a pCR patient (A) 47.47% of lymphocytes were screened, (B) 74.87% of CD3+ T cells were screened, (C) CD4+ CD8+ T cells were screened, (D) 44.76% of CD3+CD4+ T cells were screened. (E) CD3+CD8+ T cells accounted for 28.8%, (F) CD19+ B cells accounted for 10.06%, (G) CD3‐C16+CD56+ NK cells accounted for 14.04%

**FIGURE 2:**
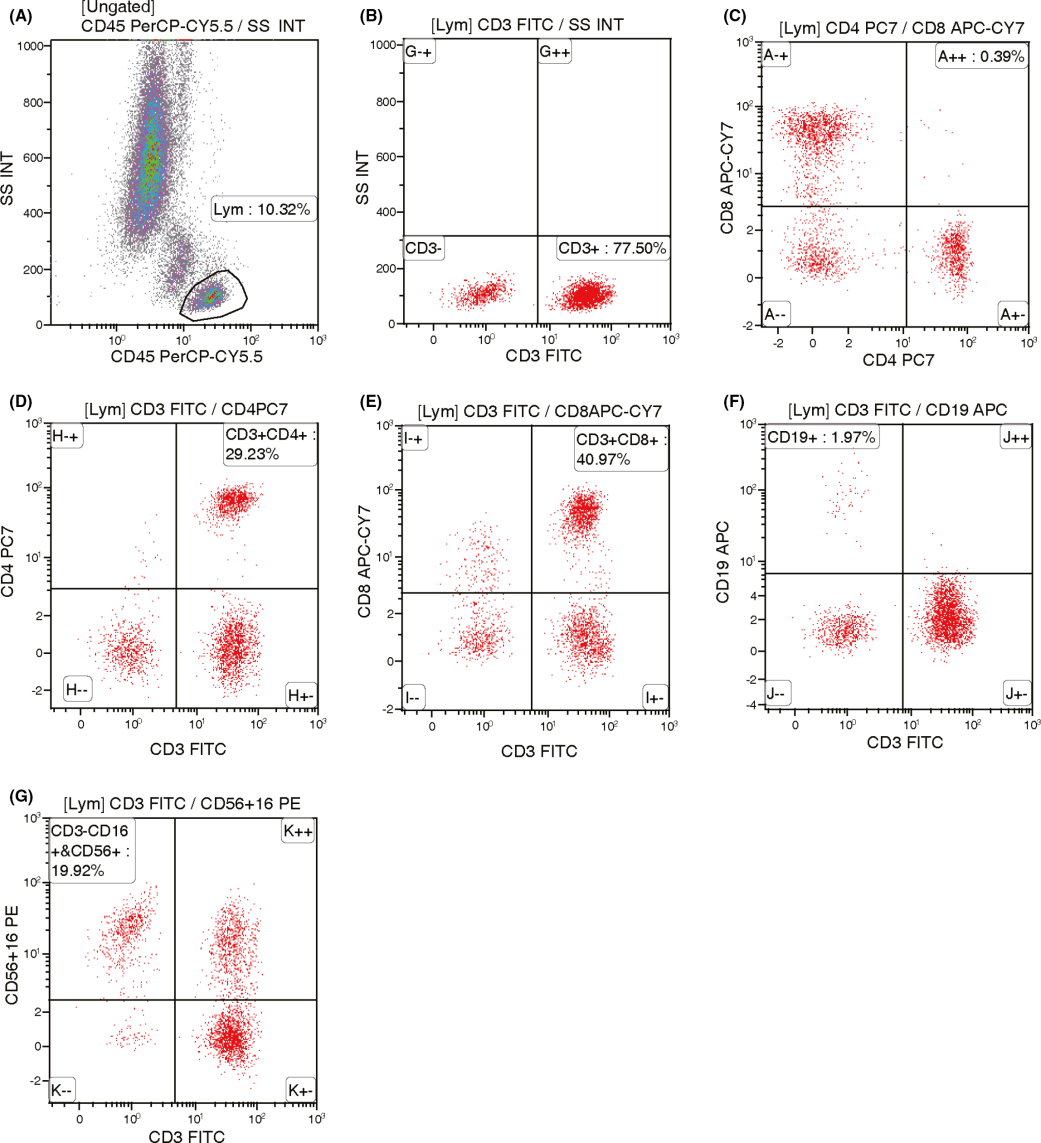
Flow cytometry of PBLs subtypes from a non‐PCR patient (A) 10.32% of lymphocytes were screened, (B) 77.50% of CD3+ T cells were screened, (C) CD4+ CD8+ T cells were screened, (D) 29.23% of CD3+CD4+ T cells were screened. (E) CD3+CD8+ T cells accounted for 40.97%, (F) CD19+ B cells accounted for 1.97%, (G) CD3‐C16+CD56+ NK cells accounted for 19.92%

Blood samples of enrolled patients within 1 week before NAT were collected, and PBLs subtypes analysis were performed according to the above methods, then the relevant distribution of CD3+, CD3+CD4+, CD3+CD8+, CD4+/CD8+ T cells, CD16+CD56+ (NK cells), CD19+ (B cells) were obtained (Figures 1, 2).

**FIGURE 3:**
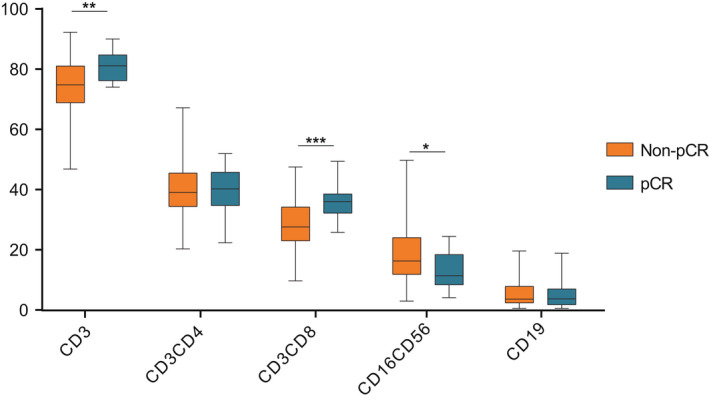
The relationship between distribution of PBLs subtypes and NAT efficacy(pCR). The abscissa represents different subtypes, and the ordinate represents the overall percentage of corresponding cells. It can be seen that CD3+ T (*p* = 0.004) cells, CD3+CD8+ T cells (*p* = 0.00028) were significantly positively correlated with pCR rate. CD16+CD56+ NK cells were negatively correlated with pCR (*p* = 0.012)

It can be seen that NK cells (*p* = 0.01) were negatively correlated with pCR rate, while CD3+ T cells(*p* < 0.01), CD3+CD8+ T cells(*p* < 0.01) were positively correlated with pCR rate, and other subtypes such as B cells (*p* = 0.71), CD3+CD4+ T cells (*p* = 0.64) showed no significant correlation with pCR rate (Figure 3).

### 3.3 Prediction effect of PBLs subtypes on efficacy of NAT

**FIGURE 4:**
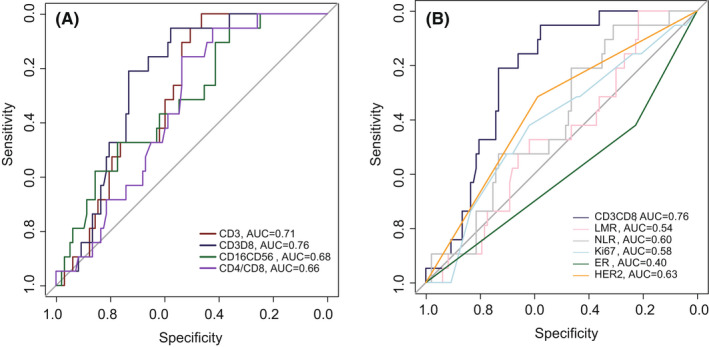
AUC curve of different indexes for pCR prediction. (A) The prediction ability of PBLs subtypes distribution for pCR, among which CD3+ CD8+ T cells were the highest (AUC = 0.76). (B) The prediction ability of CD3+CD8+ T cells and other related indicators (including LMR, NLR, Ki67, ER, PR, HER2) was further compared, and CD3+CD8+ T cells still had the highest prediction ability

**FIGURE 5:**
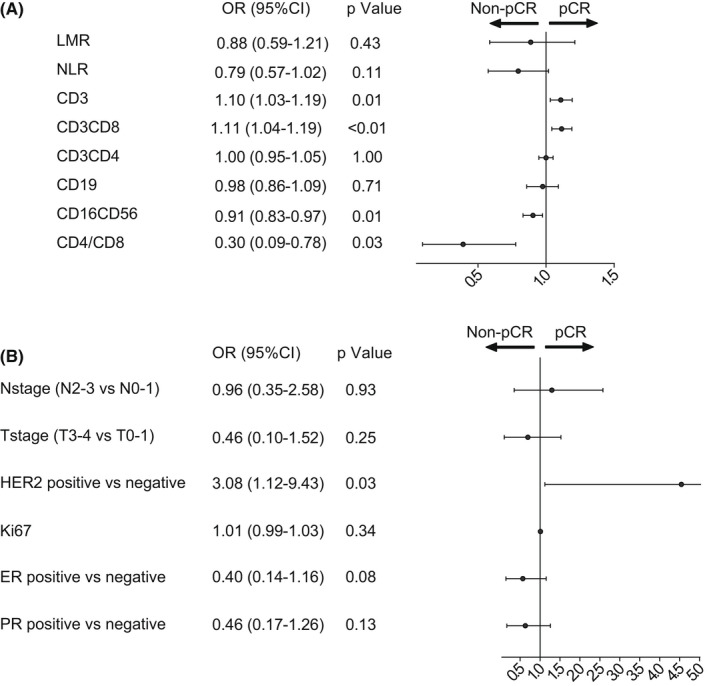
Forest plots for influence factors of pCR (A) univariate logistics regression analysis was conducted suggesting that CD3+ T cells(*p* = 0.01),CD3+CD8+ T cells(*p* < 0.01), and NK cells(*p* = 0.01) can influence pCR (B) univariate logistics regression analysis was conducted including T stage, N stage,ER,PR,HER2 expression, and Ki67

In univariate logistic regression analysis, CD3+ T cells (OR 1.10, 95% CI 1.03–1.19, *p* = 0.01), CD3+CD8+ T cells (OR1.11, 95% CI 1.04–1.19, *p* < 0.01), NK cells (OR 0.91, 95% CI 0.83–0.97, *p* = 0.01), and CD4+/CD8+ (OR0.30, 95% CI 0.09–0.78, *p* = 0.03) were found to be significant predictor of pCR (Figure 5A,B). We also evaluated the predicted accuracy of pBLs subtypes and other previously reported clinicopathological factors and found that CD8+ T cells has the largest AUC for predicting pCR (Figure 4A,B.

**FIGURE 6:**
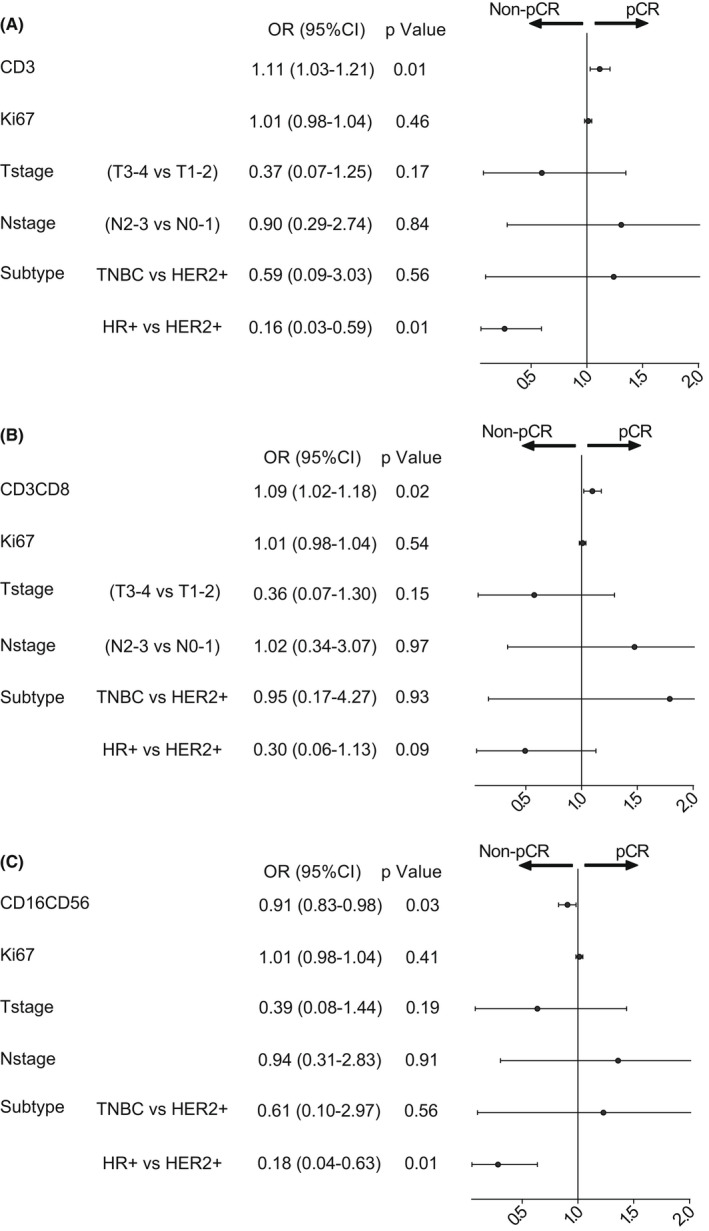
Forest plots for independent predictors of pCR (A) Multivariate logistics regression analysis was conducted with CD3+ T cells and Ki67, T stage, N stage, and subtypes, suggesting that CD3+ T cells were independent predictors (*p* = 0.01), (B) Multivariate logistics regression analysis was conducted with CD3+CD8+ T cells and Ki67, T stage, N stage, and subtypes, suggesting that CD3+CD8+ T cells were independent predictors (*p* = 0.02),(C) Multivariate logistics regression analysis was conducted with NK cells and Ki67, T stage, N stage, and subtypes, suggesting that NK cells were independent predictors (*p* = 0.03)

Multivariate regression analysis was further used, combined with other factors that may influence NAT efficacy including T stage, N stage, Ki67, BC molecular subtypes, etc. Results indicated that CD3+ T cell (OR 1.11, 95% CI 1.03–1.21, *p* = 0.01), CD8+ T cells (OR1.09, 95% CI 1.02–1.18, *p* = 0.02), and NK cells (OR 0.91, 95% CI 0.83–0.98, *p* = 0.03) were independent predictors of pCR in BC patients (Figure 6) (Table3).

**TABLE 3:** Multivariate logistics regression analysis was conducted with CD4+ T cells&CD19+ B cells and Ki67, T stage, N stage, and subtypes

		OR (95% CI)	*p*
CD4+			
CD3CD4+		1.01 (0.95–1.07)	0.72
Ki67		1.02 (0.99–1.05)	0.28
T stage		0.42 (0.09–1.46)	0.21
N stage		1.08 (0.37–3.13)	0.88
Subtypes(TNBC vs. HER2)		0.91 (0.16–4.10)	0.90
Subtypes(HR+ vs. HER2)		0.17 (0.04~0.59)	0.01
CD19			
CD19		0.95 (0.05–1.45)	0.38
Ki67		1.01 (0.99–1.04)	0.30
T stage		0.40 (0.08–1.40)	0.19
N stage		1.05 (0.36–3.05)	0.93
Subtypes(TNBC vs. HER2)		0.99 (0.18–4.46)	0.99
Subtypes(HR+ vs. HER2)		0.16 (0.03–0.57)	0.01

Abbreviation: N stage, pathological lymph nodes stage; T stage, pathological tumor stage; TNBC, triple negative breast cancer; HR+: HR+,HER2‐.

## 4 DISCUSSION

Immunity plays an important role in the prognosis and therapeutic effect of BC.^14^, ^19^ In this study, the baseline PBLs subtypes of patients receiving NAT were analyzed to explore its correlation with NAT efficacy in BC patients. The results suggested that peripheral CD3+ T cells, CD8+ T cells, and NK cells were independent predictors of pCR in BC patients. And peripheral CD8+ T cells at baseline shows highest prediction ability of pCR (AUC = 0.76). Therefore, our study can provide non‐invasive, accurate and easily accessible predictors for NAT efficacy in BC patients, and help clinicians’ better understand tumor immunity. To our knowledge, this is the first study about the relationship between PBLs subtypes and pCR in BC patients.

It is of great clinical significance to predict the NAT efficacy for BC patients. Previous studies have suggested that some clinical factors as LMR, NLR, and Ki67 can predict neoadjuvant pCR rate.^20^, ^21^, ^22^ In this study, we found that PBLs subgroups such as CD8+ T cells (AUC = 0.76), CD3+ T cells (AUC = 0.71), NK cells (AUC = 0.68), and CD4/CD8 ratio (0.66) could predict pCR probability more accurate, compared with LMR(AUC = 0.54), NLR (AUC = 0.60), and Ki67 (AUC = 0.58) in our cohort. Meanwhile, PBLs is as easy to obtain as the above index. Therefore, to some extent, our newly discovered PBLs index can replace these indexes found in previous studies and predict the efficacy of NAT in BC patients more accurately and conveniently. On the other sides, compared with traditional TILs score acquired with biopsy specimen, PBLs subtypes' test is more convenient and less invasive, and can be tested at any time during NAT, moreover, PBLs can complement the limitation of TILs in NAT efficacy prediction for BC patients, such as some BC patients do not have TILs, while PBLs can be tested in almost every single patient. To sum up, PBLs subtypes test has a certain potential for subsequent clinical application in BC patients.

Previous study indicated a significant correlation between the tumor infiltrated (TI) ‐ NK cells and the response rate of neoadjuvant‐targeted therapy combined with chemotherapy in HER2+ BC patients,^23^ while our study found that peripheral NK cells distribution was a negative independent predictor(P = 0.03) of NAT efficacy in all BC patients, independent of molecular subtypes. This contradiction may be related to the different functional status between peripheral NK cell and TI‐NK,^24^, ^25^ but the mechanism needs further study.

In BC, peripheral CD8+ T cells exist in naive form and need to be activated by dendritic cells with tumor‐specific antigen in lymphoid tissues,^26^ then those CD8+ T cells become effector T cells, which could bind to tumor cells' surface antibody, and release lytic granules to kill target cancer cells.^27^ Some studies have shown that TI‐ CD8+ T cells were associated with pCR of NAT efficacy in BC.^28^In addition, atezolizumab, a PD‐L1 inhibitor targeting CD8+ T cells, combined with chemotherapy or anti‐HER2 target therapy has a high response rate in BC treatment.^29^, ^30^, ^31^ All of these highlight the important role of TI‐CD8+ T cells in BC, but the relationship between BC peripheral CD8+ T cells and NAT efficacy remains unclear. In this study, we reported for the first time that peripheral CD8+ T cell as an independent predictor for NAT efficacy, and one possible explanation for the mechanism is that chemotherapy would kill tumor cells rather than peripheral CD8+ T cells,^32^ and lysis of tumor cells results in exposure of tumor‐associated antigens to dendritic cells, which may activating naive peripheral CD8+ T cells for further immune response and influence NAT efficacy in a reply.

However, this study has certain limitations. First, this study is a single‐center retrospective study based on a small sample size, and the results may be biased. Moreover, due to the limitation of sample size, this study did not analyze PBLs in separated molecular subgroups of BC. Second, the study adopted a relatively rough method of lymphoid subtypes analysis, we only studied total peripheral blood NK cells, while it can be further divided into different subtypes as CD56 bright NK cells and CD56 dim NK cells. Therefore, although this study indicated the relationship between PBLs subtypes and BC NAT efficacy for the first time, other more multicenter meta‐studies based on larger sample sizes are still needed to further confirm the results of this study.

## 5 CONCLUSIONS

Peripheral CD3+ T cells, CD8+ T cells, and NK cells were independent predictors of pCR in BC patients receiving NAT. Therefore, it can provide non‐invasive, accurate, and easily accessible predictors for corresponding patients, and help clinicians better understand tumor immunity.

## CONFLICT OF INTEREST

The authors declare no conflict of interest. OPEN ACCESS This article is licensed under a Creative Commons Attribution 4.0 International License, which permits use, sharing, adaptation, distribution, and reproduction in any medium or format, as long as you give appropriate credit to the original author(s) and the source, provide a link to the Creative Commons license, and indicate if changes were made. The images or other third‐party material in this article are included in the article's Creative Commons license, unless indicated otherwise in a credit line to the material. If material is not included in the article's Creative Commons license and your intended use is not permitted by statutory regulation or exceeds the permitted use, you will need to obtain permission directly from the copyright holder.

## AUTHOR CONTRIBUTIONS

Conceptualization, Jikun Feng, Xi Wang, and Feng Ye; Data curation, Jikun Feng, Xi Wang, and Feng Ye; Formal analysis, Jikun Feng; Funding acquisition, Xi Wang; Investigation, Jikun Feng; Methodology, Jikun Feng; Project administration, Jikun Feng,Xi Wang, and Feng Ye; Resources, Jikun Feng and Xi Wang; Software, Jikun Feng, Jianxia Li; Supervision, Xi Wang; Validation, Jikun Feng, xinjian Huang, Jiarong Yi, Haoming Wu, Wenjing Zhong, and Weiling Huang; Visualization, Jikun Feng; Writing – original draft, Jikun Feng; Writing – review & editing, Jikun Feng and Xi Wang.

## ETHICS STATEMENT

This study was approved by the Ethics Committee of Sun Yat‐sen University Cancer Hospital.

## Data Availability

The data that support the findings of this study are available from the corresponding author upon reasonable request.
